# Undersampling bankruptcy prediction: Taiwan bankruptcy data

**DOI:** 10.1371/journal.pone.0254030

**Published:** 2021-07-01

**Authors:** Haoming Wang, Xiangdong Liu

**Affiliations:** School of Economics, Jinan University, Guangzhou, Guangdong, China; Vellore Institute of Technology: VIT University, INDIA

## Abstract

Machine learning models have increasingly been used in bankruptcy prediction. However, the observed historical data of bankrupt companies are often affected by data imbalance, which causes incorrect prediction, resulting in substantial economic losses. Many studies have proposed the insolvency imbalance problem, but little attention has been paid to the effect of the undersampling technology. Therefore, a framework is used to spot-check algorithms quickly and combine which undersampling method and classification model performs best. The results show that Naive Bayes (NB) after Edited Nearest Neighbors (ENN) has the best performance, with an F2-measure of 0.423. In addition, by changing the undersampling rate of the cluster centroid-based method, we find that the performance of the Linear Discriminant Analysis (LDA) and Naive Bayes (NB) are affected by the undersampling rate. Neither of them is uniformly declining, and LDA has higher performance when the undersampling rate is 30%. This study accordingly provides another perspective and a guide for future design.

## Introduction

Theoretically, financial distress has varying degrees of performance [1]. Milky financial distress may express temporary cash flow difficulties, such as concepts like insolvent, default, and other images, the most serious is the business failure, or bankruptcy [2]. In most cases, the authors tend to use the final failure bankruptcy as the boundary line of distinguishing failed and non-failed companies. This business failure will make the company’s operation interruption [3]. Therefore, establishing a reliable enterprise failure prediction model is critical to the company [4].

Inaccuracy in bankruptcy forecasting can negatively impact finance and lead to a devastating blow to business owners, partners, society, and the entire national economy. Therefore, the company’s internal management, the audit, and public authorities are interested in bankruptcy prediction because it affects their decision-making. Therefore, improving the bankruptcy prediction ability seems particularly important. Based on this fact above, an increasing number of scholars use models to identify bankrupt enterprises, thereby reducing the risk to investors. From the research of Altman (1974), Beaver (1966), and Ohlson (1980) [5–7], The bankruptcy model has evolved over several decades. Ottman first created a multivariate statistical approach, using financial report data to classify the company [5]. Ohlson(1980) works for the first time to apply logic regression analysis to this field [7].

Owing to the development of economics and computer technology, not only traditional statistical models, increasingly, machine learning models are also used in this field. However, machine learning classification models are based on the assumption of two classes of data equilibrium. If there is an imbalance in the amount of data between different categories, this imbalance can interfere with the performance of machine learning algorithms. As demonstrated by Kubat(1997) [8], bankruptcy prediction is a typical problem in unbalanced data classification and prediction. Data imbalance processing methods can be divided into data level, algorithm-level cost-sensitive learning, and hybrid approaches. The algorithm-level modification makes the original model more suitable for category forecasting. Among them, allocating misclassification costs to correct class prediction is the most popular algorithm [9]. Another hybrid method involves combining the preprocessing step with the algorithm, which significantly improves the classification performance. Data preprocessing is simpler and more efficient than the first two. The idea is to change the size of the training data space by adding or deleting samples in the data space to reduce the discriminatory behavior of the unbalanced data.

Data preprocessing is a quick and efficient method for processing imbalance issues, and oversampling is widely concerned because of its versatility. However, undersampling is worth more attention. In this study, we use the publicly available data set of bankrupt Taiwanese companies and choose the evaluation index, F2-measure, which is suitable for the unbalanced data set after considering its data characteristics. Then, by sampling different linear and nonlinear models, including Support Vector Machine (SVM), Logistic Regression (LR), Linear Discriminant Analysis (LDA), Gaussian Naive Bayes (NB), XGBoost (XGB), Neural Networks (NN), k-Nearest Neighbors (KNN), Random Forest (RBF), those with performances exceeding the baseline are screened out, NB, NN, and LDA algorithms are filtered out. After that, five undersampling methods are used for three chosen models to combine the model with the best performance. Finally, the combination of ENN and NB achieves the best classification performance. In addition, by changing the sampling rate, the change of the F2-measure is observed, indicating that the underwent can improve the model’s performance as the possible means of data preprocessing (change in the sampling rate affects the performance of the model). This result indicates that finding a suitable undersampling rate improves the performance of modes while maintaining optimal information and speed is possible.

The main contributions are as follows:

1. In this study, we used a systematic framework, which including three steps of (1)using the most suitable metric so that we can evaluate models, (2)quickly testing the classifiers, (3) tuning the best models. Most bankruptcy scenarios are suitable in this framework.
2. According to the characteristics of the data, we select the most appropriate metric for the unbalanced bankruptcy data, F2-measure. This idea is reasonable for the bankruptcy data set, but there are few optimization adjustments to the metrics according to the data characteristics in other bankruptcy prediction studies.
3. By changing the undersampling rate, we discuss the performance changes of the two models that exceed the baseline model’s F2-measure scores. The model can maintain higher performance in a range of undersampling rates.

The rest of this article is as follows: the first part, literature review, provides an overall assessment of bankruptcy predictions and the undersampling method of data preprocessing, and the next section describes the methodologies used in this study, the experimental results of the proposed method are provided in the part of experimental results, and the final piece is analysis and summary.

## Literature review

**Fig 1:**
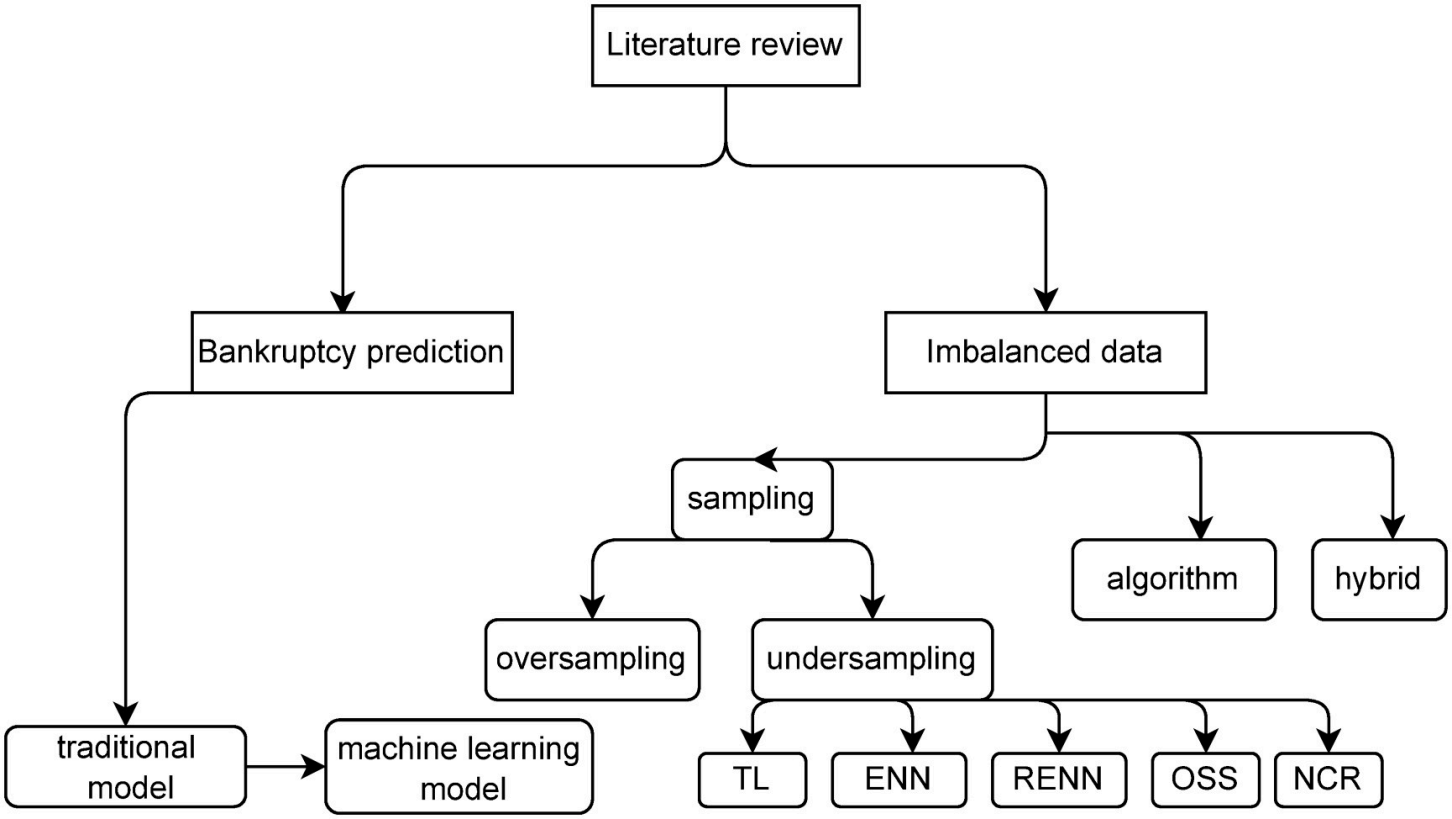
Organization figure. Structure of literature review.

The structure of the review can be clearly seen in Fig 1 below.

### Important literature in the field of bankruptcy prediction

In 1966, Beaver focused on a single ratio and used various ratios to measure a company’s capabilities. His study provides investors with 30 ratios that illustrate key relationships [6]. Altman’s Z-score model, which was proposed in 1968, was first used for bankruptcy prediction; later, five variables were selected to generate the Z-score model [5]. Ohlson (1980) used the logit model to select data from 1970 to 1976 to analyze the four factors that affect enterprise bankruptcy. Since then, an increasing number of models have been used for bankruptcy prediction for decades [7].

Balcaen and Ooghe (2006) summarised the development of different models and found that univariate analysis does not require complex statistical knowledge because it requires strict prerequisites, and the risk index model sets a number of different weighting ratios to calculate the score [10].

Taffler and Agarwal (2007) tested the predictive ability by Z-scores and found that the Z-score model was a good choice in most cases; however, it was necessary to develop new models based on specific situations [11]. Some statistical models can then be used for bankruptcy prediction, such as univariate analysis and MDA multi-classification analysis [12].

Currently, with the wide application of machine learning models in various fields, [13–16]. The machine learning model plays an important role in bankruptcy classification. However, most machine learning algorithms for classification assume that the instances of each class are equal.

In addition to linear models, neural network-based methods have also been used to predict the bankruptcy of different firms [17, 18]. Durica(2019) used a decision tree to predict the financial situation of Polish companies; precisely, a classification and regression tree and a chi-square automatic interaction detector were used to obtain valid results [19].

There has been considerable research on the choice of financial indicators as well as the inclusion of other indicators, such as financial structure-related indicators [20]; deep learning of image data has also been used to predict bankruptcy [21]. The combination of financial ratios FRS and corporate governance metrics CGI is used in 2016 to improve the predictive performance of SVM, KNN, NB, CART, and MLP [22].

Bankruptcy prediction models are more and more combining with a machine learning model based on the other fields, such as using the financial ratio to improve the prediction ability [22]. However, few studies focus on the combination of the undersampling method.

### Literature on the data processing of imbalanced data

The industry has widely used oversampling since it was proposed by Chawla et al. (2002) [23]. Although undersampling reduces the amount of information in the data, it helps to obtain the same number of class samples and makes the training phase faster.

The condensed nearest-neighbor (CNN) is an early data-cleaning technique, and the random sample selection method of the CNN-compressed nearest neighbor can be modified. This method is effectively used to eliminate overlapping in the sampling method [24].

The nearest-neighbor rule has been used to reduce the number of pre-classified samples. This Edited Nearest Neighbors (ENN) method has been continuously improved [25], and the work of Tomek (1976) is a further improvement on this method [26]. Moreover, ENN is often combined with other methods, such as the neighborhood cleaning rule (NCR) based on ENN [27]. This method deletes two of the three nearest neighbors. In addition, SMOTE is also used in combination with ENN and Tomek links [28]; therefore, it can be said that the Tomek link and ENN are the most classical undersampling methods.

One Sided Selection (OSS) is an undersampling technique used to improve the two methods mentioned above. It was proposed by Kubat(1997). This method uses Tomek links first, followed by the US-CNN. Tomek links are used as an undersampling method to remove most instances of noise and borders [8].

The Neighbor Cleaning Rule (NCR) is a method that combines a CNN to delete redundant instances and ENN to delete noisy instances. Compared with the previous OSS, this method focuses more on retaining the quality of instances in most classes [29]. It uses ENN to identify all instances in the majority class, and after processing, a single-step version of CNN is used to delete instances in the majority class, which is more than half the number of the minority class.

Altincay(2004) used a combination of undersampling based on clustering and Adaboost [30]. In addition to this study, many other studies have also adopted sub-sampling based on clustering and fine-tuning on the basis of this idea [31, 32].

## Research methodology

**Fig 2:**
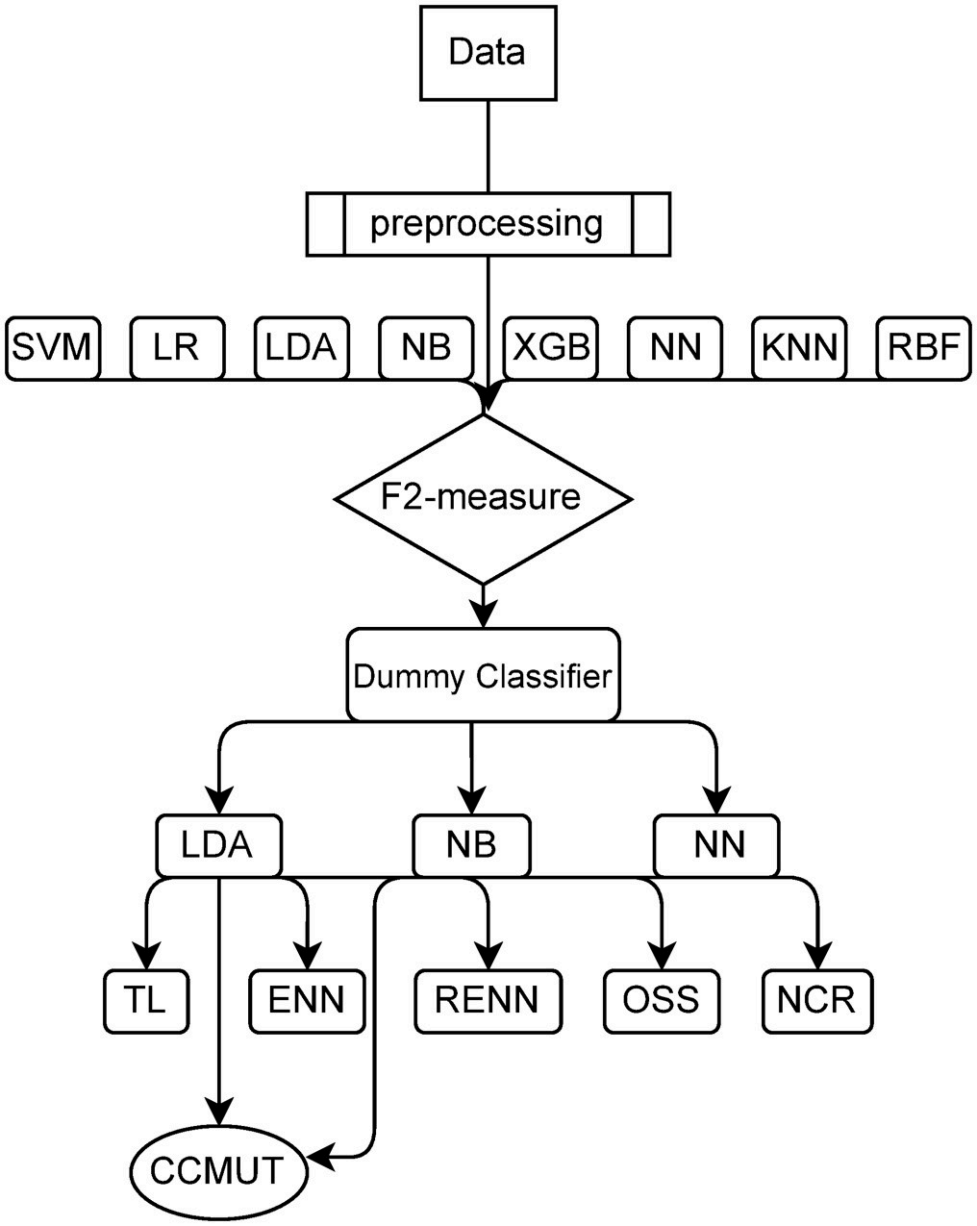
Experimental procedure. Complete experimental flow chart.

Fig 2 shows the process of the entire experiment. The process in this study consists of five parts: the first part of this section describes the data sources; the second part describes the data preparation; the third part describes the selection of evaluation indicators, and the fourth and fifth parts describe the sub-sampling methods and selection model.

### Data source

**Fig 3:**
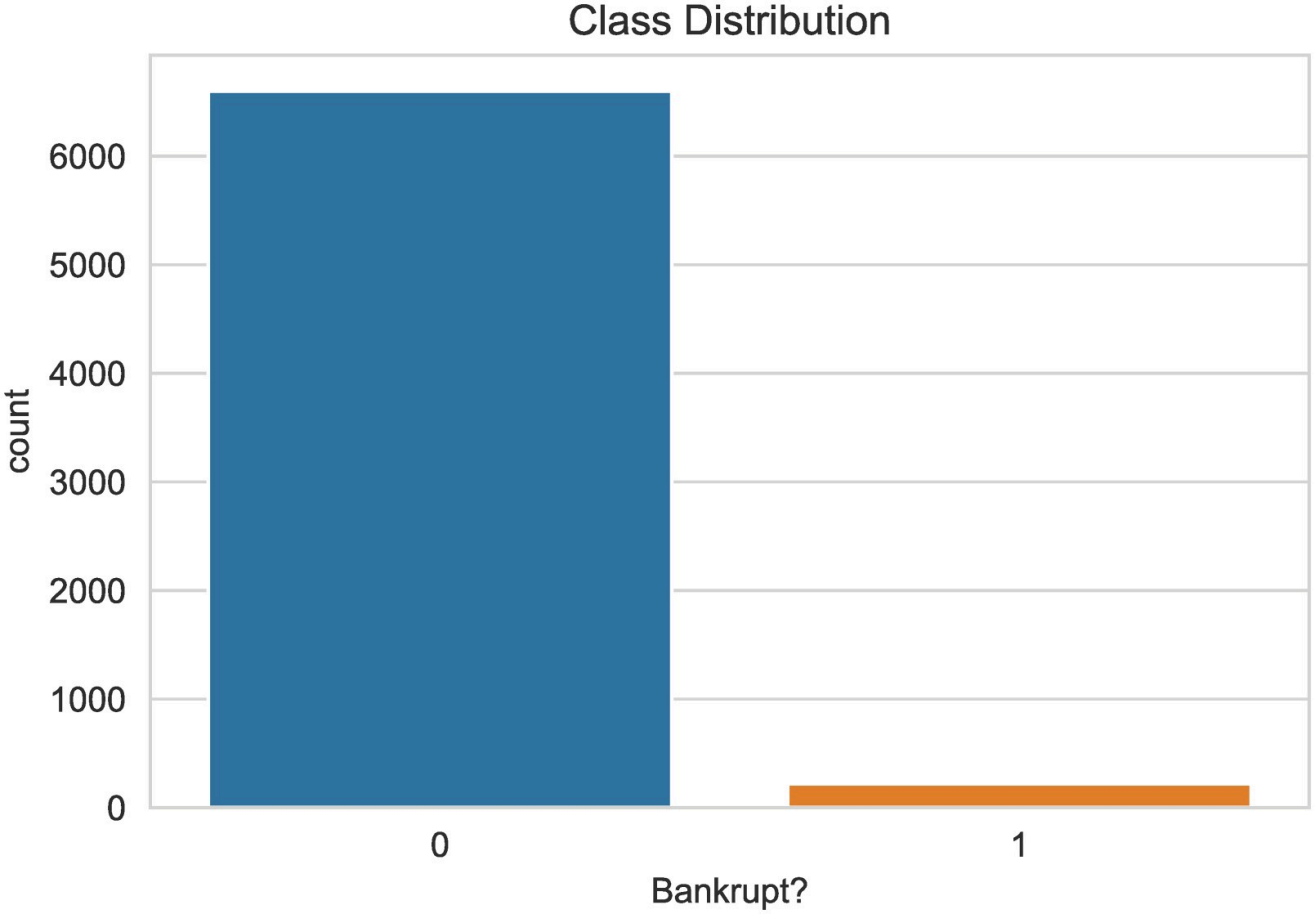
Class distributioin. As can be seen from the graph, there is a significant imbalance in the data, with 97% majority and 3.226% minority.

Taiwan’s bankruptcy data were obtained from the Taiwan Economic Journal from 1999 to 2009. Corporate bankruptcy was defined based on the business rules of the Taiwan Stock Exchange. It contains 6819 enterprises, 96 attributes, two categories. Fig 3 illustrates the huge imbalance with 96.774% non-bankruptcy enterprises and 3.226% bankruptcy enterprises. Bankruptcy and non-bankruptcy firms are marked as ‘1’ and ‘0’ separately. The data source is from https://www.kaggle.com/fedesoriano/company-bankruptcy-prediction.

### Data preprocessing

Checking for missing values and negative values before the study is necessary to ensure the reliability of the data. Table 1 summarizes that both are 0.

**Table 1:** Anomaly records.

**N/As**:	0
**Negative values**:	0

**Fig 4:**
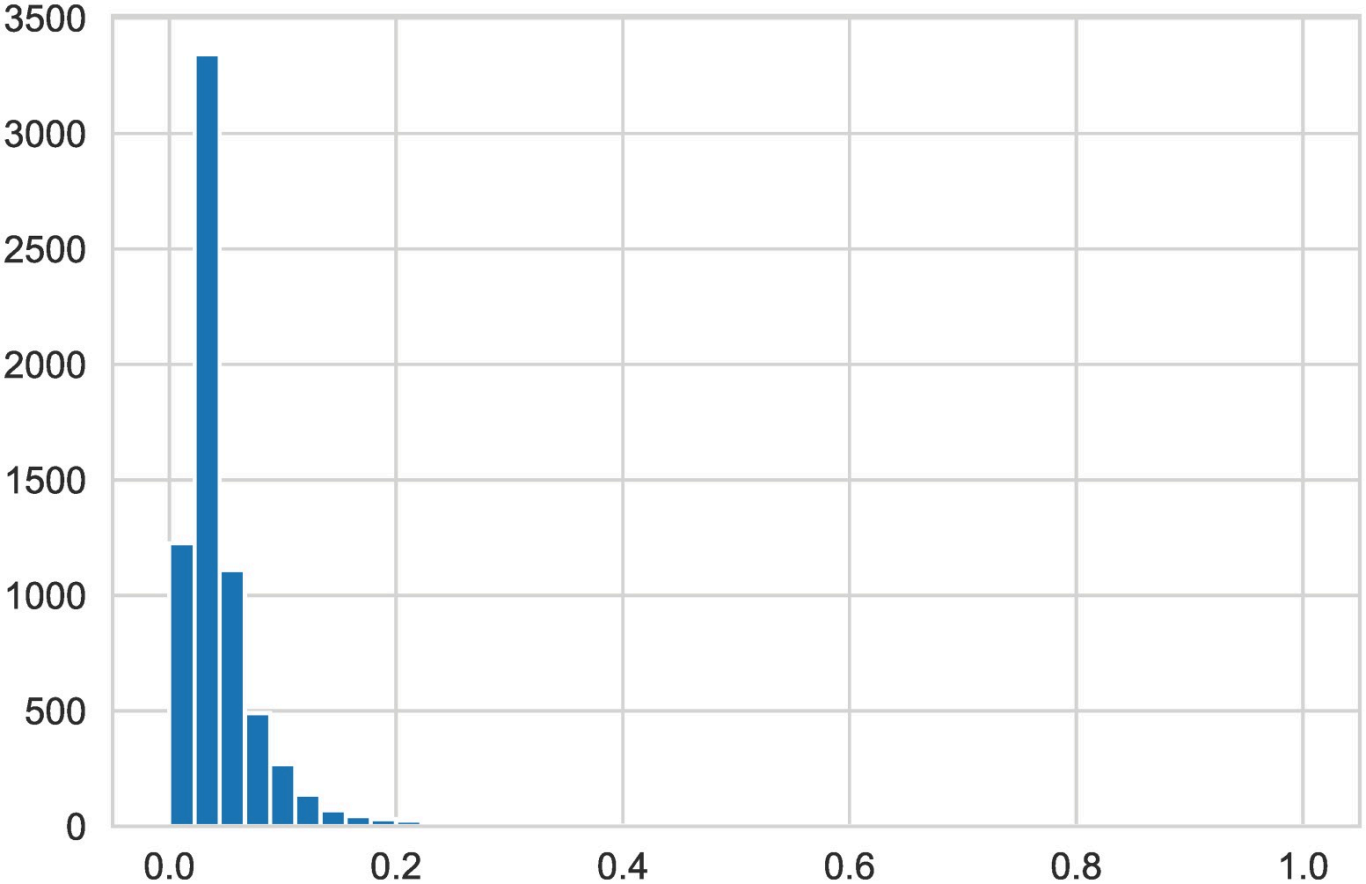
’Equity to Liability’. The histogram of this attribute value is skewed to some extent.

**Fig 5:**
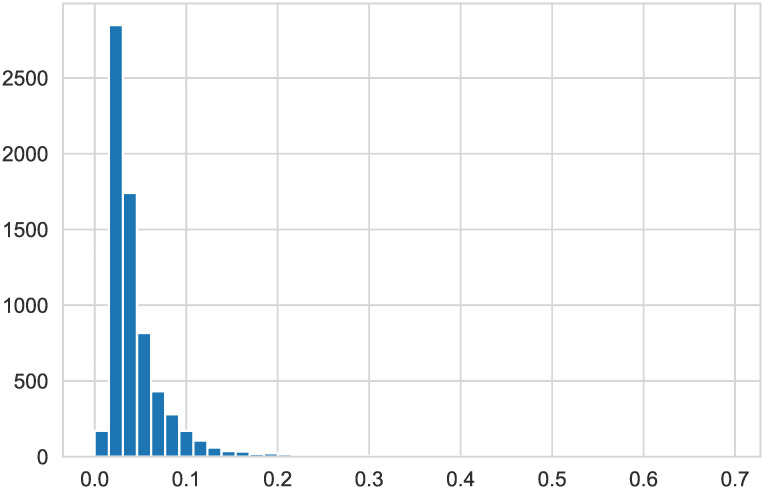
’Equity to Liability’. A spot check of the ‘Equity to Liability’ skew value reveals that it has reached more than 7.40.

When plotting all variables, it can be seen from Figs 4 and 5 that some variables have skewness; therefore, logarithmic transformation is performed on all data to reduce their skewness.

**Fig 6:**
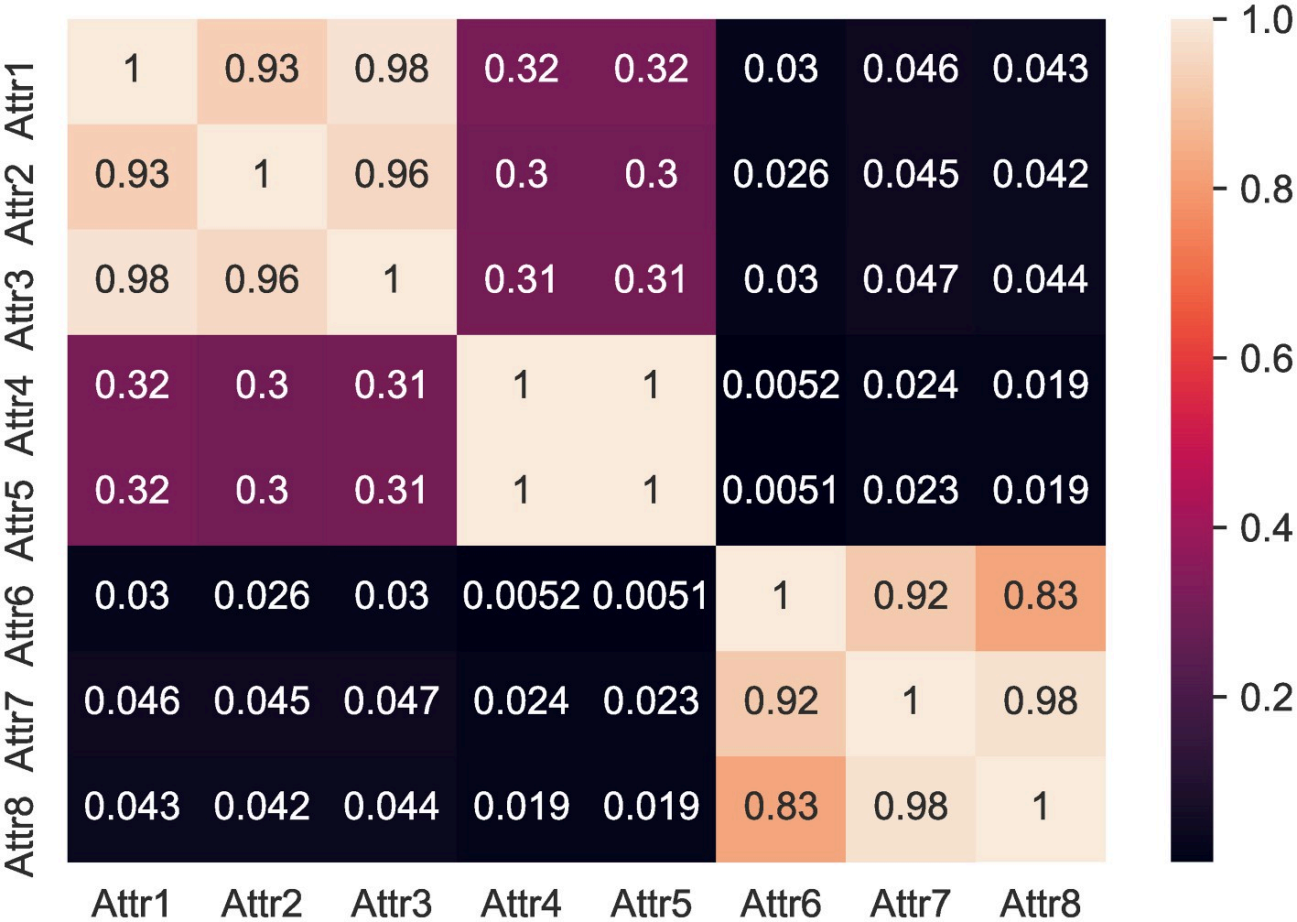
Heatmap of some variables. Although there is a strong correlation of 0.93 between Attr1 and Attr2, 0.98 between Attr1 and Attr3, and 0.92 between Attr6 and Attr7, most of the variables have weak correlation.

As can be seen from Fig 6, which shows the Persson diagram of some variables, there is no solid correlation between the variables, and no feature screening was performed in this study to preserve the integrity of the input information.

After the log transformation, using MinMaxScaler within each fold of cross-validation, the pipeline is used to avoid data leakage.

### Evaluation metrics

The most widely used is the classification accuracy, Eq (1), belonging to the threshold metric. The use of standard metrics in imbalanced domains can lead to sub-optimal classification models and might produce misleading conclusions since these measures are insensitive to skewed parts [33].

$$\begin{array}{c} Accuracy=\frac{\text{TP}+\text{TN}}{\text{TP}+\text{FP}+\text{TN}+\text{FN}}. \end{array}$$ (1)

Table 2 is a confusion matrix with negative classes, ‘0’ and positive classes, ‘1’, each specific name is summarized in this table. In this study, the minority is positive class, bankrupt companies, and vice versa.

**Table 2:** Binary confusion matrix.

	Positive Prediction	Negative Prediction
**Positive Class**	True Positive(TP)	False Negative(FN)
**Negative Class**	False Positive(FP)	True Negative(TN)

There is 96.774% of the majority class of in this dataset. Therefore, if each instance is classified into the majority class, the accuracy will be as high as 96.774%, resulting in an imbalance in the traditional accuracy index. To avoid this imbalance inaccuracy when comparing different algorithms, choosing a reasonable evaluation index is necessary.

We aim to select the most suitable evaluation index according to the characteristics of the Taiwan bankruptcy dataset. Precision can quantify the number of correct positive predictions,

Precision and recall have different focuses; therefore, precision is sensitive to data distribution but not to recall. Eqs (2) and (3) show these two metrics. The F1-measure, which combines these two evaluation indicators, is a widely used metric, evaluating the performance of algorithms dealing with data imbalances, but in this dataset, the false-negative class is more costly. It should be modified to match the characteristics better. The formula for the F1-measure is as follows:

$$\begin{array}{c} Precison=\frac{\text{TP}}{\text{TP}+\text{FP}} \end{array}$$ (2)

$$\begin{array}{c} Recall=\frac{\text{TP}}{\text{TP}+\text{FP}} \end{array}$$ (3)

$$\begin{array}{c} F_{1}=\frac{\text{Precision}\times \text{Recall}}{\text{Precision}+\text{Recall}} \end{array}$$ (4)

The essence is the case where beta in Fbeta-measure is 1, and the formula of Fbeta-measure is as follows [34]:

$$\begin{array}{c} F_{\beta }=\frac{(1+\beta^{2})\times \text{Precision}\times \text{Recall}}{\beta^{2}\times \text{Precision}+\text{Recall}} \end{array}$$ (5)

In this study, we set the beta of F-measure to 2, which is intended to minimize false negatives. If a bankruptcy prediction is predicted to be non-bankrupt, this confusion can lead investors to trust a poorly-run business blindly. Thus, we need to minimize this by using the F2-measure metric.

### Undersampling methods

The methods used in this study are classical undersampling methods. These methods have been mentioned in the literature. Tomek Links (TL), Edited Nearest Neighbors (ENN), Repeated Edited Nearest Neighbors (RENN), One Side Selection (OSS), and Neighbor Cleaning Rule (NCR) did we select as undersampling methods. Besides, the Centroid-based Majority Undersampling Technique (CMUT) was an additional method and compared by changing the undersampling rate.

TL, Tomek Links can be summarized in a nutshell:” instances a and b define a Tomek Link if: (i) instance a’s nearest neighbor is b, (ii) instance b’s nearest neighbor is a, and (iii) instances a and b belong to different classes.” [34]. This mind for finding TL can ensure that boundary and noisy instances will have nearest neighbors [34].

ENN, Edited Nearest Neighbors Rule uses k = 3 nearest neighbors locate the misclassified examples in the dataset and then deleting them before applying the K = 1 classification rule. This method of resampling and classification was proposed by Dennis Wilson in 1972.

RENN is a repeated version for the ENN method, as can be seen from its name that it is based on the ENN since RENN is an endless repetition of ENN editing. However, the steps will be automatically terminated after a certain number of repetitions [26].

OSS is a technique that combines with Tomek Links and Condensed Nearest Neighbor Rule. This method was proposed by Kubat [8]in 1997, and the overview of the procedure is obtained from the article, Addressing The Curse of Imbalanced Training Sets: One-sided Selection:

1. Let S be the original training set.
2. Initially, C contains all positive examples from S and one randomly selected negative example.
3. Classify S with the 1-NN rule using the examples in C, and compare the assigned concept labels with the original ones. Move all misclassified examples into C that is now consistent with S while being smaller.
4. Remove from C all negative examples participating in Tomek links. This removes those negative examples that are believed borderline and/or noisy. All positive examples are retained. The resulting set is referred to as T.

Neighborhood Cleaning Rule uses CNN to delete redundant examples, looking for a subset of the sample set that does not result in no loss of model performance, followed by ENN, deleting noisy instances.

The centroid-based majority undersampling technique uses the concept of feature space geometric clustering to classify the importance and non-importance of instances. Clustering is a method used for unsupervised learning; however, CMUT only uses the concept of finding cluster centroids, where the point closest to the cluster centroids can be considered the most important. Thus, instances away from most class centroids can be deleted, and the number of deleted examples can be controlled by the undersampling rate, which makes it possible to adjust the undersampling rate to obtain the best performance.

Euclid’s formula is as follows:

$$\begin{array}{c} \text{Euclidean}\;\text{distance}\left(p,q\right)=\sqrt{\sum_{i=1}^{n}{\left(q_{i}-p_{i}\right)}^{2}} \end{array}$$ (6)

*p* and *q* represent the attribute value in the sample, *N* is the number of instances and 1 < *i* < *N*.

**Algorithm 1** The centroid-based majority undersampling technique

1: finding the cluster centroid

2: calculating the Euclidean distance from the cluster centroid to each sample

3: arranging samples indices in descending order of distance

4: deleting the instances

5: returning the undersampled Majority Matrix

### Prediction models

To avoid the extreme situation when the training set and test set are separated, this experiment uses repeated hierarchical 10-fold cross-validation. Each fold has approximately 600 examples; each fold is nearly 96% of the non-bankruptcy companies, and 3% is bankruptcy. 10-fold cross-validation repeats three times, which means each model fits 30 times to avoid a fluke. It is necessary to take measures to avoid artificially high scores caused by data leakage. This study ensured that only the training set information was used in the data processing by using ‘pipeline’ and carefully check the fit and transform process.

It is worth mentioning that many researchers have encountered data leakage problems during cross-validation because they have carried out various types of preprocessing on the data before dividing the dataset, and many scholars are not aware of this part in their data processing.

Santos(2018) found that this type of data leakage significantly improves the results compared to the original dataset [35]. However, this is not reasonable because we cannot know any information about the test set in advance, which will be ensured in this study; all preprocessing will use only the training set information.

To quickly find a predictive model suitable for the dataset, it is prevalent to simultaneously perform spot checks on linear and nonlinear models. By comparing with the F2-score of DummyClassifier, all other models can be compared simultaneously. Here, the parameter of DummyClassifier is set to a constant of 1, which is the baseline model in this study.

The algorithms used are Support Vector Machine (SVM), Logistic Regression (LR), Linear Discriminant Analysis (LDA), Gaussian Naive Bayes (NB), XGBoost (XGB), Neural Networks (NN), k-Nearest Neighbors (KNN), Random Forest (RBF). These models are all widespread supervised machine-learning models in bankruptcy prediction.

## Experimental results

**Fig 7:**
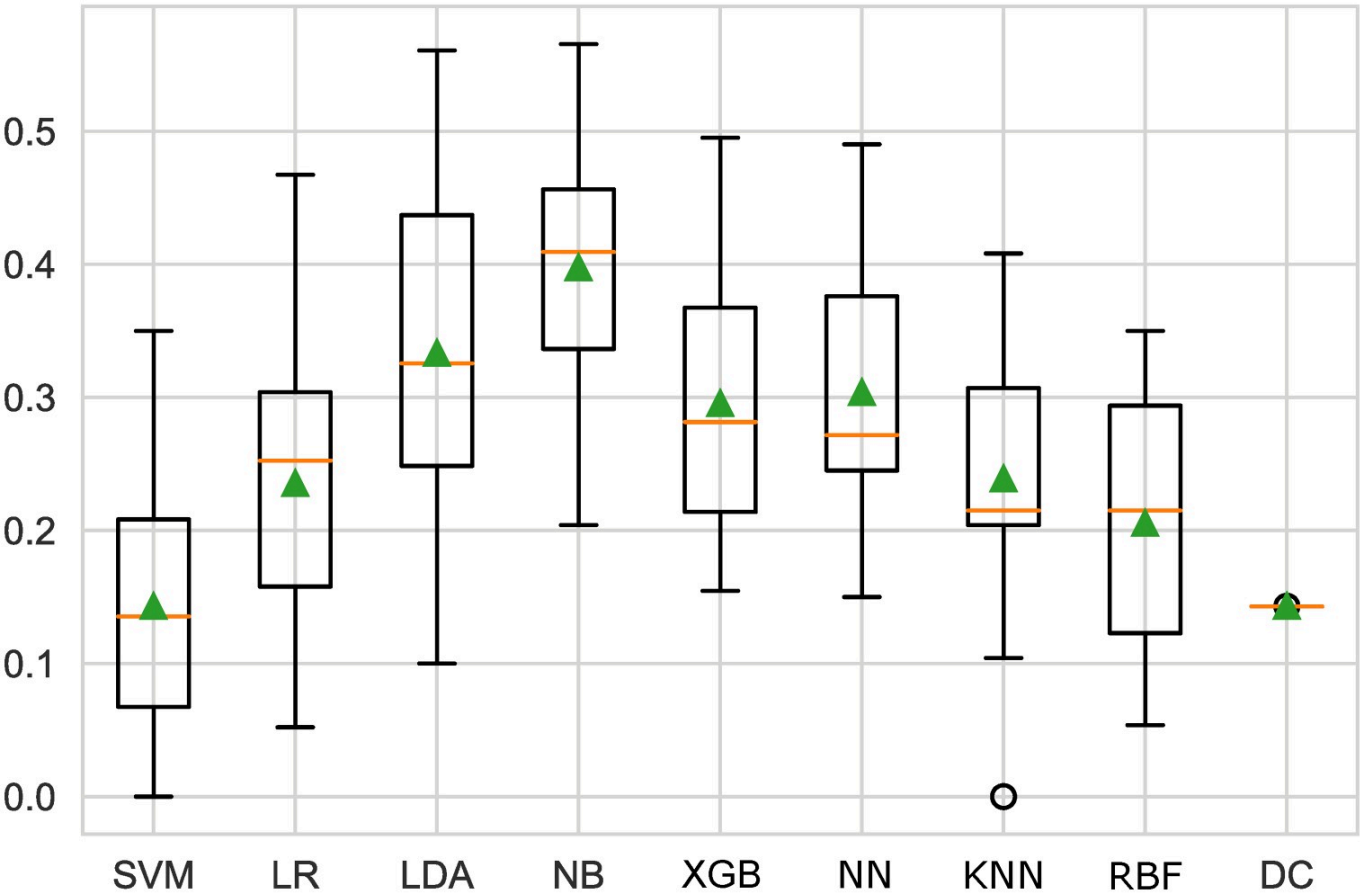
Boxplot of different models. Among all the models, NB had the best F2-score, followed by LDA with an F2-measure of 0.333; LR, XGB, and NN had almost the same score (0.235, 0.295, and 0.304, respectively); and SVM had the lowest score, which was equal to the baseline score.

Fig 7 illustrates that both linear discriminant analysis and naive Bayes and neural networks provide an F2-score higher than the baseline classifier—Dummy Classifier score F2: 0.143. The exact values can be seen in Table 3.

**Table 3:** F2-measure of machine learning models.

Models	mean(F2-measure)	std(F2-measure)
**SVM**	0.143	0.89
**LR**	0.235	0.96
**LDA**	0.333	0.122
**NB**	0.398	0.086
**XGB**	0.295	0.090
**NN**	0.304	0.094
**KNN**	0.239	0.088
**RBF**	0.205	0.086
**DC**	0.143	0.000

**Fig 8:**
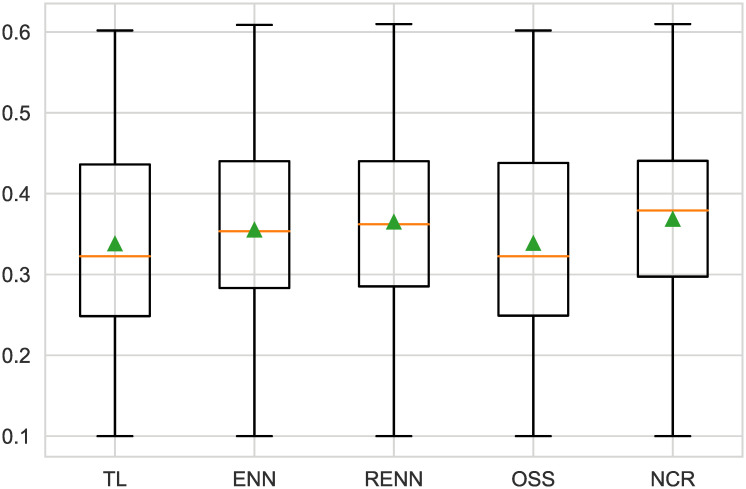
Boxplot of undersampling for LDA. All five sampling methods had similar scores; however, NCR had the lowest standard deviation.

Undersampling helps to remove sample points from most classes along the decision boundary, and as can be seen in Fig 8, all models increase their predictive performance when linear discriminant analyses are undersampled in a variety of ways. The NCR method gives the highest F2-score. Detail is in Table 4.

**Table 4:** F2-measure of LDA.

Models(LDA)	mean(F2-measure)	std(F2-measure)
**TL**	0.338	0.124
**ENN**	0.355	0.126
**RENN**	0.365	0.123
**OSS**	0.338	0.124
**NCR**	0.368	0.122

**Fig 9:**
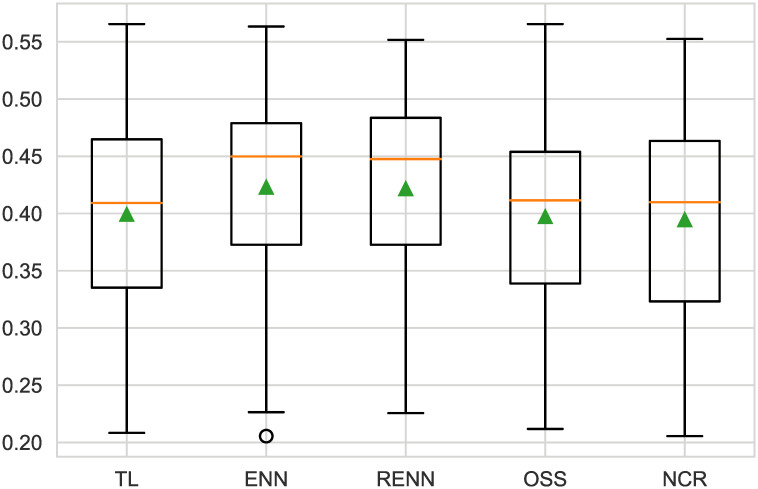
Boxplot of undersampling for NB. The ENN score was the highest (0.423), the RENN score was slightly lower (0.079), and the other three undersampling scores were similar.

For the original naive Bayesian model with the best performance, the ENN and RENN resulted in the most improvement, as shown in Fig 9. The ENN has the best effect for the NB model, while the RENN has better stability than ENN. Table 5 shows the Numerical value.

**Table 5:** F2-measure of NB.

Models(NB)	mean(F2-measure)	std(F2-measure)
**TL**	0.399	0.086
**ENN**	0.423	0.084
**RENN**	0.421	0.079
**OSS**	0.397	0.085
**NCR**	0.394	0.086

**Fig 10:**
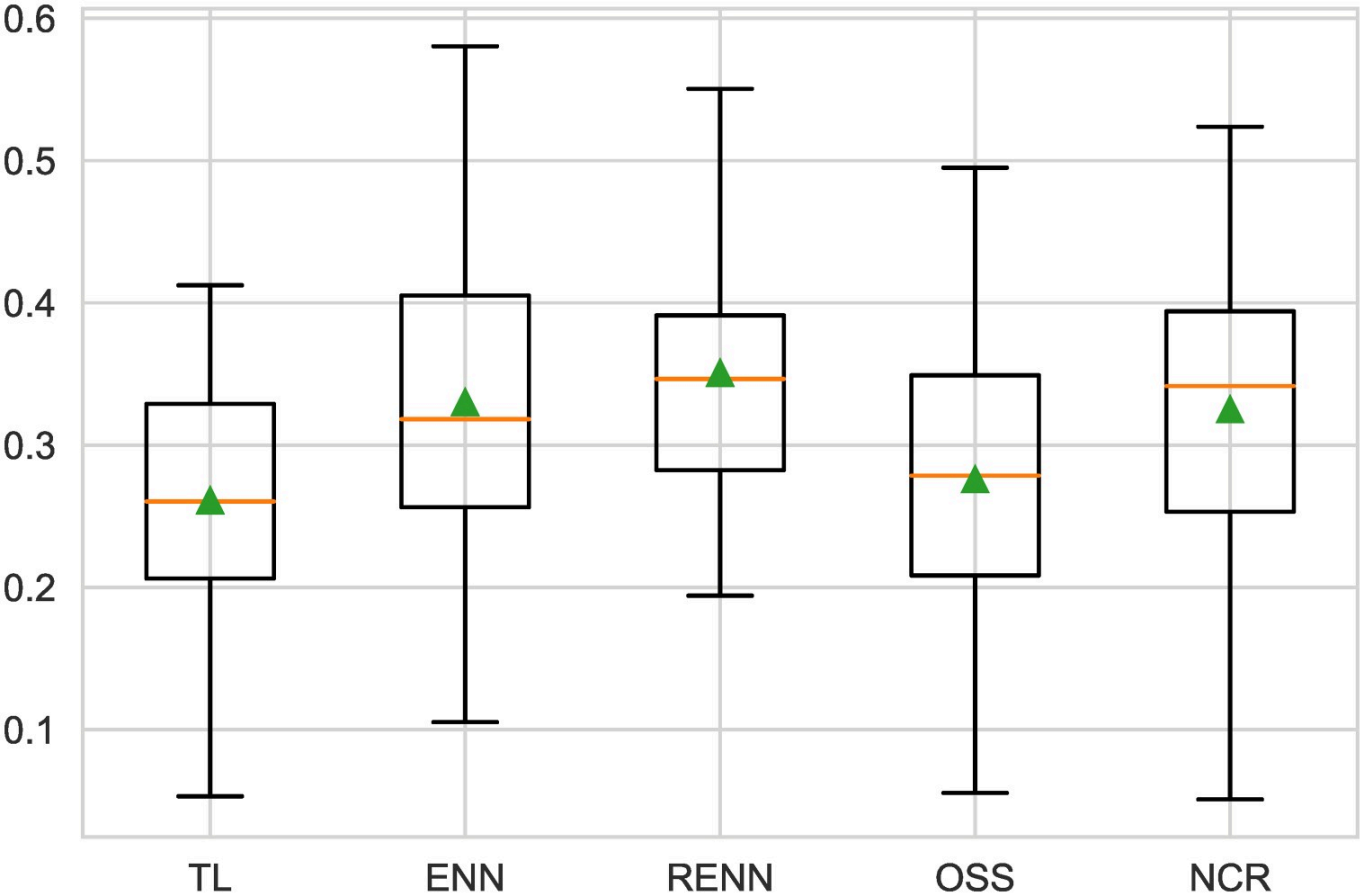
Boxplot of undersampling for NN. The ENN RENN OSS shared similar highest scores; however, the standard deviation was not low, and the RENN score was 0.350.

For the neural network model with weak interpretation ability, compared with the original 0.304, the results were improved after undersampling, except the Tomek link and OSS. All the comparisons are in Fig 10.

**Fig 11:**
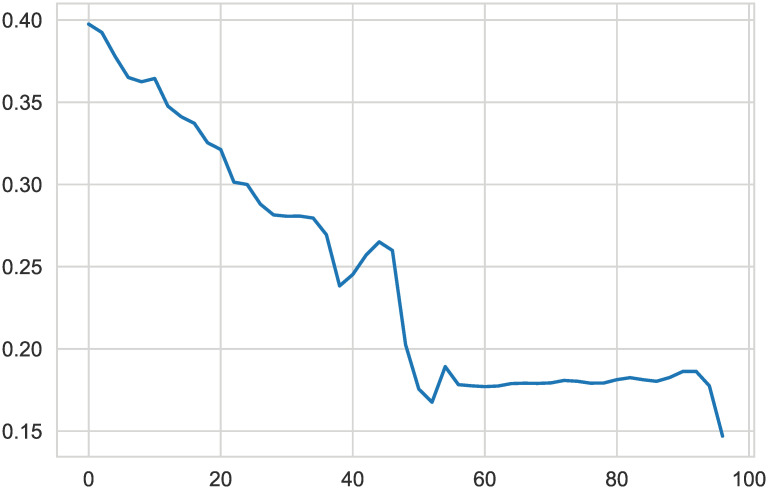
Centroid undersampling for NB. Without undersampling, the highest F2-score is 0.3975. Continuously increasing the undersampling rate does not blindly reduce the evaluation score. It is non-decreasing but increases when the undersampling rate is approximately 54% and 80%.

Undersampling of the clustering idea was used for the NB model. As shown in Fig 11, by changing different undersampling ratios, the evaluation score was changed.

**Fig 12:**
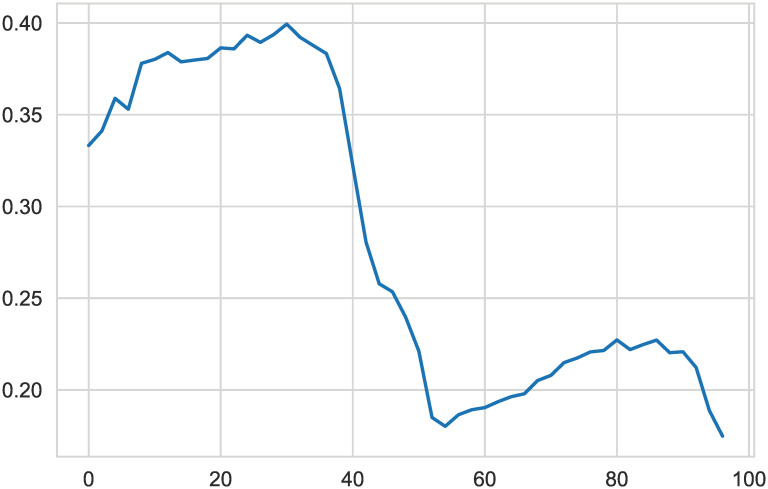
Centroid undersampling for LDA. LDA has a higher F2-measure when the undersampling is 30%, with a value of 0.3994, and has the lowest value at 54%; it is non-decreasing but increases after 54%.

Fig 12 indicates that the changes in the undersampling rate can affect the LDA model.

## Discussion

Evaluating a mixture of machine learning models on the data, the linear discriminant analysis, naive Bayes, and neural network work better, deserving further attention.

After undersampling, the traditional linear discriminant analysis method is improved among the five under-sampling methods, but the NCR method had the worst stability, and the Naive Bayes method has the best prediction effect. In combination with the ENN under-sampling method, maximum improvement is obtained. Compared with the RENN algorithm, the ENN algorithm has better performance, but their F2-measure is almost the same, and the RENN algorithm has more stability and lower standard deviation.

In addition, although the neural network can not provide the highest F2-measure, the results of the model are also improved in the case of undersampling. With the complexity of the network structure and the simplification of the data preprocessing data, the neural network’s effectiveness seems to have improved, and the RENN method provides the most remarkable improvement. But their standard deviation is not low. Therefore, LDA and NB are selected for CMUT, and their F2-measure could be altered by changing undersampling rates. Although the effect is not as good as that of ENN’s NB model, for NB, the change of undersampling rate does not further improve performance. As for LDA, when the undersampling rate is 30%, LDA achieves a better prediction result than that without preprocessing, reaching 0.3994.

## Conclusion

In our study, a three-step framework is used to analyze Taiwan data. First, considering the characteristics of the data set, we use the F2-measure, which adjusts the beta of the F1-measure to evaluate the machine learning model by repeated cross-validation, fully considering the data leakage. By comparing the F2-score of different Machine learning models, we spot-check and choose the three most promising classification models. On this basis, in the third stage, five undersampling methods are used to optimize the algorithms. We find that the NB after ENN has the best performance, and the F2-measure is 0.423. The performance of the LDA and NB models is affected by the undersampling rate. This fact shows that in a specific range, finding the undersampling rate which can improve the performance of the model is a feasible method of data preprocessing. It is feasible to improve the model performance in future work by increasing the undersampling rate while keeping the optimal information and speed.
